# *Gemmata algarum*, a Novel Planctomycete Isolated from an Algal Mat, Displays Antimicrobial Activity

**DOI:** 10.3390/md22010010

**Published:** 2023-12-21

**Authors:** Gaurav Kumar, Nicolai Kallscheuer, Mohammad Kashif, Shabbir Ahamad, Uppada Jagadeeshwari, Sreya Pannikurungottu, Tom Haufschild, Moses Kabuu, Chintalapati Sasikala, Christian Jogler, Chintalapati Venkata Ramana

**Affiliations:** 1 Department of Plant Sciences, School of Life Sciences, University of Hyderabad, P.O. Central University, Hyderabad 500046, India; kgrv021@gmail.com (G.K.);; 2 Department of Microbial Interactions, Institute of Microbiology, Friedrich Schiller University, 07743 Jena, Germany; 3 Cluster of Excellence Balance of the Microverse, Friedrich Schiller University, 07743 Jena, Germany; 4 Bacterial Discovery Laboratory, Centre for Environment, Institute of Science and Technology, JNT University Hyderabad, Kukatpally, Hyderabad 500085, India

**Keywords:** *Gemmataceae*, *Planctomycetota*, bioactivity, budding, wetland bacteria, agar plate assay

## Abstract

Axenic cultures of two strains, JC673^T^ and JC717, both belonging to the phylum *Planctomycetota*, were isolated from distinct geographical locations in India. Strain JC673^T^ was obtained from algal mats of a wetland situated in the state of Kerala, India, while strain JC717 originated from the Central Marine Fisheries Research Institute (CMFRI), state of Tamil Nadu, India. The two strains share 99.9% 16S rRNA gene sequence similarity and are most closely related to *Gemmata obscuriglobus* UQM 2246^T^ (99.3% 16S rRNA gene sequence identity). The newly isolated strains are Gram-negative, grow aerobically and tolerate up to 4% (*w*/*v*) NaCl and a pH of up to 9.0. Cells are spherical and form pink-pigmented colonies. The respiratory quinone is MK-6. Major fatty acids are C_18:0_, C_16:1_ω5c and C_16:0_. Polar lipids include phosphatidylcholine, phosphatidylethanolamine, several unidentified amino lipids, unidentified phospholipids, additional unidentified lipids, and an unidentified choline lipid. The polyamine spermidine is produced by the two strains. The strains have a genome size of about 8.2 Mb with a DNA G+C content of 67.6%. Solvent-based culture extracts of the isolates showed antimicrobial activity against three bacterial test strains. Their phylogenetic position along with differences in morphological, physiological, and genomic features support the classification as a new species of the genus *Gemmata*, for which we propose the name *Gemmata algarum* sp. nov. Strain JC673^T^ (=KCTC 72851^T^ = NBRC 114340^T^) and JC717 are the type and non-type strain of the new species, respectively.

## 1. Introduction

Currently, less than 1% of bacterial diversity is covered by axenic cultures [1] and the remaining 99% of elusive microbes belong to the ‘microbial dark matter’ as they are so far recalcitrant to any cultivation attempt [2]. Untargeted cultivation approaches to unearth fastidious bacterial members residing in the same ecosystem along with fast-growing members appear like the search for a polar bear in a snowstorm. Hence, to unearth novel elusive microbes belonging to understudied bacterial phyla such as *Planctomycetota*, targeted cultivation and deep-cultivation approaches are required. Members of the phylum *Planctomycetota* occur ubiquitously and receive attention due to various cell biological peculiarities. Previous studies have suggested that model strains of the phylum are able to import intact high-molecular-weight polysaccharide molecules like dextran into an enlarged periplasmic space for further degradation into oligosaccharides and subsequently to monosaccharides [3]. This ‘selfish’ strategy (in comparison to the secretion of polysaccharide catabolic enzymes) is of advantage in oligotrophic environments, in which easily degradable sugars are absent and carbon needs to be obtained from the breakdown of, e.g., plant- or algal-derived polysaccharides. Additional cell biological peculiarities include an uncharacterized form of asymmetric cell division (“budding”) and the lack of otherwise canonical bacterial cell division proteins [4,5]. In silico analyses of the genomes of various *Planctomycetota* members underline their potential to produce novel small molecules with potential antimicrobial and other health-promoting activities [6]. Recent studies demonstrated that metabolite extracts from members of the phylum indeed have antimicrobial activities [7,8]. Hence, the phylum can be regarded as an untapped source of novel bioactive compounds.

*Planctomycetia*, the currently largest class of the phylum, contains the order *Gemmatales* with the sole family *Gemmataceae*. This family is currently formed by the following nine genera: *Fimbriiglobus*, *Frigoriglobus*, *Gemmata*, *Limnoglobus*, *Telmatocola*, *Tuwongella*, *Urbifossiella*, *Zavarzinella*, and *Thermogemmata* [9,10,11,12,13,14,15,16,17,18]. The currently largest explored genus of the family is *Gemmata* with the three described species *G. obscuriglobus*, *G. massiliana*, and *G. palustris.* The respective type strains were isolated from freshwater, hospital water and peat, respectively [9,16,19].

Recent studies have shown that India harbours a rich planctomycetal diversity which is reflected by the isolation of several axenic cultures from different aquatic and terrestrial habitats [20,21,22,23,24]. In this study, we describe a novel species belonging to the genus *Gemmata* based on two novel strains that were independently isolated from two distinct and distantly located ecosystems (ca. 600 km distance). Strain JC673^T^ was isolated from an algal mat on the surface of the wetland in the village Pallikkara, Kerala (south-western part of India). This wetland occurred due to the influx of seawater (backwater) from the Arabian Sea. The Kerala backwaters form a network of brackish lagoons and canals lying parallel to the Arabian Sea of the Malabar coast. The other isolate, strain JC717, was isolated from an algal mat growing on the wall of a building of the CMFRI (Central Marine Fisheries Research Institute), Mandapam, Tamil Nadu (South-eastern part of India).

Based on the results of a polyphasic characterisation approach, we propose to classify strain JC673^T^ as the type strain of a novel species within the genus *Gemmata* for which we propose the name *Gemmata algarum* sp. nov. Strain JC717 is an additional member of the novel species. For comparative taxonomic studies in the laboratory, its current closest relative *G. obscuriglobus* DSM 5831^T^ (=UQM 2246^T^) was obtained from the DSMZ, Germany.

## 2. Results and Discussion

### 2.1. Phylogenetic Inference

**Figure 1:**
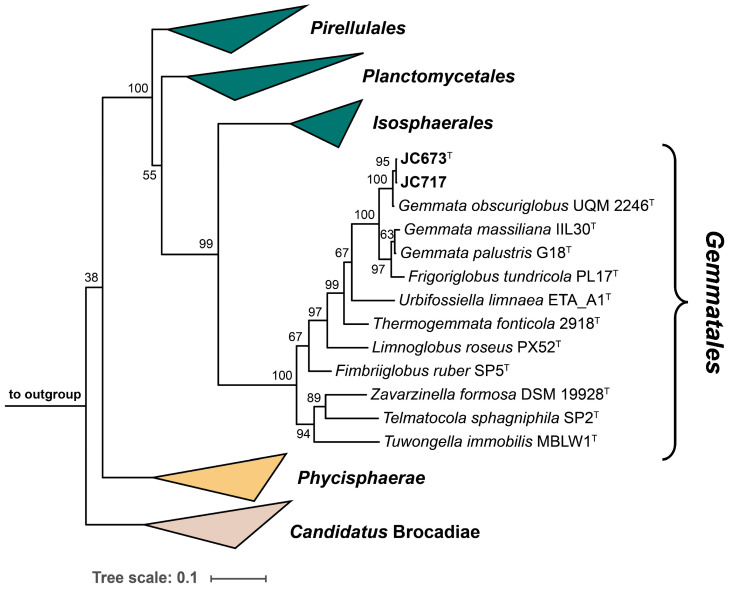
**Maximum likelihood 16S rRNA gene sequence-based phylogenetic tree.** The phylogenetic tree based on 16S rRNA gene sequences shows the phylogenetic relationship of strains JC673^T^, JC717 and other closely related members of the order *Gemmatales*. FastTree was used for tree reconstruction with 1000 bootstrap replications (given at the nodes, in %) and *Opitutus terrae* (NCBI acc. no. AJ229235), *Kiritimatiella glycovorans* (acc. no. NR_146840) and *Lentisphaera araneosa* (acc. no. NR_027571), served as outgroup. Bar, 0.1 nucleotide substitutions per position.

**Figure 2:**
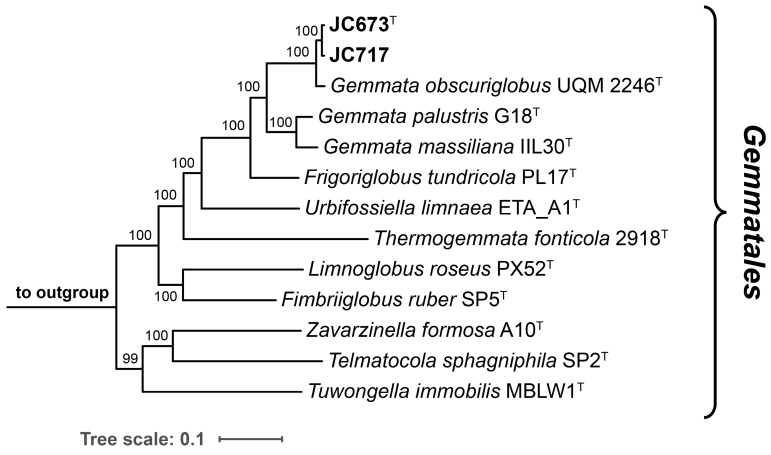
**Multi-locus sequence analysis (MLSA)-based phylogenetic tree.** The maximum likelihood phylogenetic tree based on MLSA shows the position of the two here described strains in relation to their closest described neighbours of the order *Gemmatales*. The tree was computed based on a set of 30 single-copy protein-coding genes in a maximum likelihood approach with 500 bootstrap replications using the tool autoMLST (see material and methods section for details). Bootstrap values are given at the nodes (in %). The genomes of *Rhodopirellula baltica* SH1^T^ (GenBank acc. no. BX119912.1), *Pirellula staleyi* DSM 6068^T^ (acc. no. CP001848.1) and *Blastopirellula marina* DSM 3645^T^ (acc. no. GCA_000153105.1) served as outgroup. Bar, 0.1 nucleotide substitution per position.

The 16S rRNA genes of strains JC673^T^ and JC717 have a sequence length of 1496 bp and differ at two nucleotide positions. A nucleotide BLAST analysis of the sequences yielded the three characterized *Gemmata* species as current closest relatives. This finding is in line with the position of the two strains in the two maximum-likelihood phylogenetic trees constructed based on 16S rRNA gene sequences and multi-locus sequence analysis (MLSA) (Figure 1 and Figure 2). Surprisingly, *G. palustris* G18^T^ clustered with *Frigoriglobus tundricola* PL17^T^ in the 16S rRNA gene sequence-based tree, which would render the genus paraphyletic (Figure 1). This, however, appears to be a known issue related to the 16S rRNA sequences of the two strains (G18^T^ and PL17^T^) that was also observed in the phylogenetic tree presented in the species description publication of *G. palustris* G18^T^ [9]. The unexpected clustering appeared despite a 16S rRNA gene sequence similarity of <95% for comparison of *F. tundricola* PL17^T^ with the characterized species of the genus *Gemmata*. This value justified the delineation of strain PL17^T^ from the genus *Gemmata* in the original species description manuscript although the AAI value turned out to fall above the genus threshold [13]. In the more reliable MLSA-based trees presented in the same publication and in this study, *F. tundricola* PL17^T^ clusters on a separate branch, thereby ensuring the monophyletic clustering of the *Gemmata* members (Figure 2) [25]. In general, the comparison of clustering patterns in phylogenetic trees constructed based on different marker genes can be inconclusive. In any case, the analysis of the phylogenetic position of novel isolates should be supplemented by the analysis of phylogenetic markers with consideration of the proposed threshold values for species or genus delineation and by phenotypic differences obtained during the polyphasic characterization.

**Figure 3:**
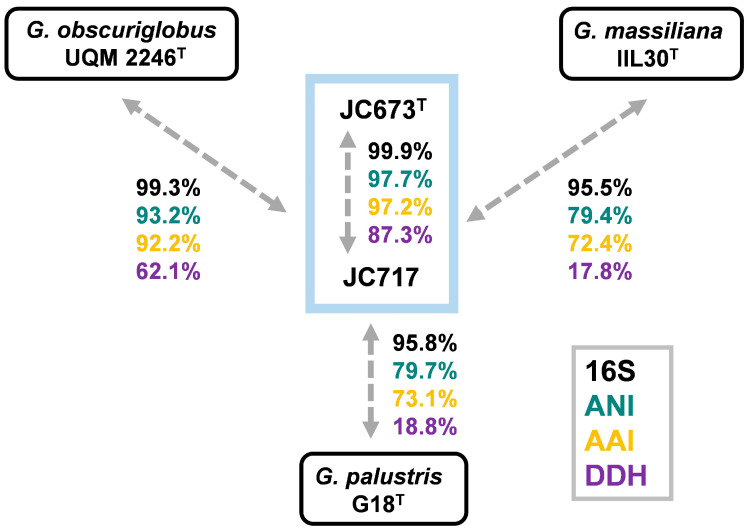
**Comparison of phylogenetic markers.** Methods used: 16S rRNA gene sequence identity (16S), average nucleotide identity (ANI), digital DNA-DNA hybridization (dDDH) and average amino acid identity (AAI).

A BLAST analysis of the nearly identical 16S rRNA gene sequences of strains JC673^T^ and JC717 revealed similarity values of 99.3%, 95.5% and 95.8% with *G. obscuriglobus* UQM 2246^T^, *G. massiliana* IIL30^T^ and *G. palustris* G18^T^, respectively (Figure 3). This is an indication for relationship on the level of the same genus. An analysis of other commonly used phylogenetic markers for the comparison with the three *Gemmata* species yielded similarity values in the range of 79.4–93.2% (ANI), 17.8–62.1% (dDDH), and 72.4–92.2% (AAI). Except for 16S rRNA gene sequence similarities, the obtained values for all analysed markers fell below the recommended species threshold values of 95% (ANI), 70% (dDDH) and 94% (AAI), respectively [25,26,27,28]. Hence, these results support the assumption that the two novel isolates belong to a distinct species of the genus *Gemmata*. High values for dDDH (87.3%), ANI (97.7%), and AAI (97.2%) obtained during comparison of the novel isolates indicates that both belong to the same species (Figure 3).

### 2.2. Genomic Characteristics

The draft genomes of the strains JC673^T^ and JC717 have a size of 8.20 Mb and 8.25 Mb with N50 values of 94,033 and 68,100, respectively. The genomes are at least 1 Mb smaller compared to the other current members of the genus whereas the DNA G+C content of 67.6% (for both strains) is the highest in the current genus and closer to *G. obscuriglobus* UQM 2246^T^ than to the other two members of the genus (Table S1). The automated genome annotation yielded 6505 and 6668 genes for strains JC673^T^ and JC717, respectively, of which 6364 and 6542 are protein-coding genes. In strain JC717, 95 genes code for RNAs (87 genes for tRNAs, 2 genes for 5S rRNAs, 1 gene each for 16S rRNA and 23S rRNA and 4 for non-coding RNAs). In strain JC673^T^, 88 genes code for RNAs (81 genes for tRNAs, 1 gene each for 5S rRNAs, 16S rRNA and 23S rRNA and 4 for non-coding RNAs) (Table S1).

**Figure 4:**
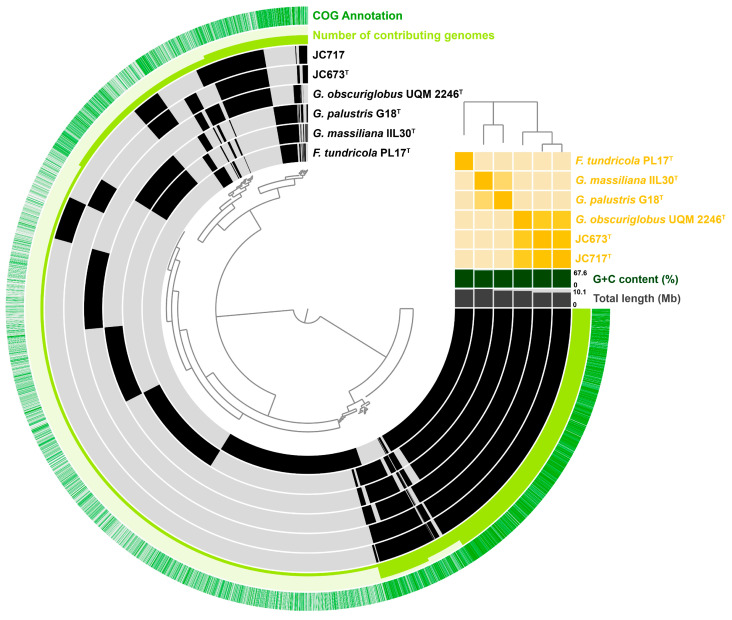
**Pangenome of strains JC673^T^ and JC717 along with current closest relatives.** Each open circle represents the pangenome of a strain that is coloured black when the gene is present in the respective genome. The tree in the upper right corner reflects the relatedness of the strains based on average nucleotide identity (ANI) values.

An OrthoVenn2 analysis showed that strains JC673^T^ and JC717 share 3836 orthologous clusters with *G. obscuriglobus* UQM 2246^T^, *G. massiliana* IIL30^T^, and *G. palustris* G18^T^. In addition, 41 clusters turned out to be exclusively absent in strain JC673^T^ and 49 clusters in JC717 when compared with other members of genus *Gemmata* (Figure S1). These results could be confirmed by the analysed pangenome of the current members of the genus *Gemmata* (Figure 4). In addition to the core genome shared by all analysed strains (3–5 o’clock in the pangenome visualization), the two here analysed strains share additional genes with *G. obscuriglobus* UQM 2246^T^ (11 o’clock in the visualized pangenome). On the other hand, genes absent in the two isolates and *G. obscuriglobus* UQM 2246^T^ are shared by the other two type strains (10 o’clock in the pangenome) (Figure 4). These results are in accordance with the close relation of the novel isolates to *G. obscuriglobus* UQM 2246^T^ and the more distant relation to *G. massiliana* IIL30^T^ and *G. palustris* G18^T^ (that in turn are more related to each other, cf. ANI-based tree in the pangenome).

### 2.3. Genome-Based Analysis of Primary and Secondary Metabolic Features

The genomes of strains JC673^T^ and JC717 code for all enzymes required for the activity of central metabolic pathways of chemoorganoheterotrophic bacteria, namely glycolysis, tricarboxylic acid (TCA) cycle, pentose phosphate pathway, and oxidative phosphorylation. Like other members of the genus, the strains harbour most of the genes responsible for chemotaxis, including *cheB*, *cheR*, and *cheY*. Like other members of the phylum *Planctomycetota*, the strains lack the cell division gene *ftsZ*. An HMM-based search for environmental bio-element families showed the absence of genes coding for nitrogen-fixing proteins in strain JC673^T^ and all other members of the genus *Gemmata*, except for *G. palustris* G18^T^. Although *nirB* genes (coding for the large subunit of nitrite reductase) were found, the gene encoding the small subunit (*nirD*) and the functional markers for denitrifiers, *nirK* and *nirS*, were not detected in any member of the genus. A putative selenate reductase, large subunit of arsenite oxidase and some of the genes involved in sulphite oxidation were also detected in the genome of strain JC673^T^. All strains belonging to genus *Gemmata* along with strains JC673^T^ and JC717 harbour putative gene clusters for biosynthesis of bioactive compounds. The predicted enzyme classes include non-ribosomal peptide synthetase (NRPS)-like enzymes, polyketide synthases (HgIE-class, type III, and mixed HgIE-type I) and enzymes involved in isoprenoid/terpenoid biosynthesis. However, genes putatively involved in the production of resorcinol-derived compounds were predicted exclusively in *G. massiliana* IIL30^T^ and *G. palustris* G18^T^ (Figure S2).

### 2.4. Morphological and Physiological Analyses

**Figure 5:**
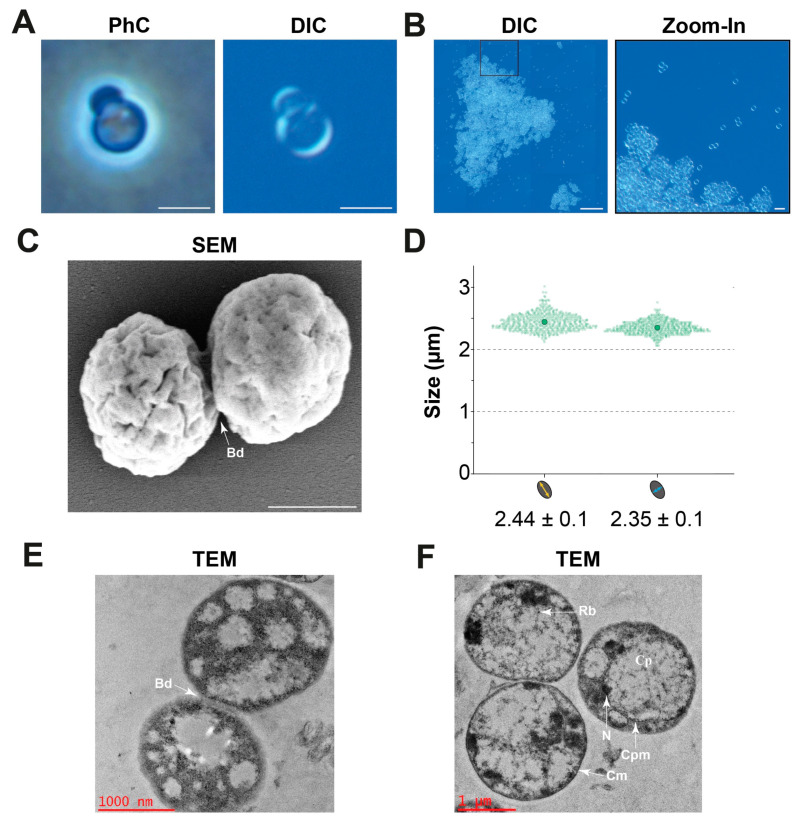
**Light and electron microscopy of strain JC673^T^**. (**A**) Phase contrast (PhC) and differential interference contrast (DIC) light microscopy show a round to slightly oval cell morphology and asymmetric cell division (budding). Scale bars represent 2 µm. (**B**) Strain JC673^T^ can form large aggregates. Scale bars represent 50 µm or 5 µm (zoomed-in image). (**C**) SEM micrograph depicting the late stage of cells dividing via asymmetric cell division (budding, Bd), the scale bar represents 1000 nm. (**D**) Cell size determination. (**E**,**F**) TEM images depicting the planctomycetal cell plan; cytoplasmic membrane (Cpm), cell membrane (Cm), ribosome (Rb), and condensed nucleoid (N).

The cell morphology was analysed using light and electron microscopy. Due to the high degree of similarity, the analyses were only performed for the type strain JC673^T^. Cells of the strain have a spherical to slightly oval-shaped cell morphology and divide by asymmetric cell division (budding, Figure 5A). They can form small aggregates with only a few cells or larger aggregates of multiple thousand cells that can reach a diameter of up to 320 µm (Figure 5B). SEM micrographs show the presence of crateriform structures on the entire cell surface (Figure 5C). Cells of strain JC673^T^ have a cell size of 2.44 µm × 2.35 µm (Figure 5D). TEM images (Figure 5D,E) show the late stage of asymmetric division (budding, Bd). Additional TEM images (Figure 5E) visualized the typical planctomycetal cell plan with cell membrane (Cm), cytoplasmic membrane (Cpm), cytoplasm (Cp), condensed nucleoid (N), and ribosomes (Rb).

A comparative characterisation of physiological and chemotaxonomic features was performed with strain *G. obscuriglobus* UQM 2246^T^ (ordered as DSM 5831^T^ from the DMSZ) as the current closest neighbour of strains JC673^T^ and JC717. *N*-Acetylglucosamine was an optional substrate for all three strains when other sources of carbon (glucose) and nitrogen were present in the culture medium. The following compounds were used as carbon and energy source by all three strains: α-d-glucose, sucrose, mannose, d-xylose, and lactose. Neither of the strains utilized the following substrates: mannitol, sodium propionate, inositol, sodium succinate, malate, sorbitol, benzoate, citrate, and sodium pyruvate. Ascorbate, starch, fructose, maltose, and galactose were only utilized by strains JC673^T^ and JC717. Fumarate utilization was exclusive for *G. obscuriglobus* UQM 2246^T^. All tested strains utilized the following nitrogen sources for biomass formation: ammonium sulphate, peptone, yeast extract, glycine, l-serine, sodium nitrate, l-glutamine, and l-glutamate. Neither of the strains utilized the following nitrogen sources: l-arginine, l-lysine, l-leucine, cysteine, and l-tyrosine. The following nitrogen sources were exclusively utilized by the strains JC673^T^ and JC717: l-isoleucine, methionine, l-phenylalanine, and l-proline. However, l-histidine and dl-threonine were exclusively utilized by *G. obscuriglobus* UQM 2246^T^ and nitrate was reduced exclusively by this strain (Table 1). In case that an additional source of sodium is present, NaCl is not obligate for growth of *G. obscuriglobus* UQM 2246^T^, strain JC673^T^, and strain JC717. NaCl concentrations (*w*/*v*) of 1%, 3%, and 4% were tolerated by the strains, respectively. All three strains showed activity for alkaline phosphatase, esterase (C4), esterase lipase (C8), leucine arylamidase, naphthol-AS-BI-phosphohydrolase, and acid phosphatase and gave negative results for the activity of lipase (C14), trypsin, α-galactosidase, cysteine arylamidase, β-glucuronidase, mannosidase, *N*-acetyl-β-glucosaminidase, and α-fucosidase. Valine arylamidase, β-galactosidase, α-glucosidase, and β-glucosidase activities are exclusively present in *G. obscuriglobus* UQM 2246^T^. α-Chymotrypsin activity was exclusively present in strains JC673^T^ and JC717 (Table 1).

### 2.5. Chemotaxonomic Characterisation

The major fatty acids in strains JC673^T^ and *G. obscuriglobus* UQM 2246^T^ are C_18:0_, C_16:1_ω5c, and C_16:0_. In terms of fatty acid composition, minor differences were found among the two compared strains (Table S2). The identified major polar lipids for the strains are phosphatidylcholine (PC) and phosphatidylethanolamine (PE). However, several unidentified amino lipids (AL1-3), unidentified phospholipids (PL1-3), other unidentified lipids (UL1-5), and an unidentified choline lipid (CL) are present (Figure S3). The major polyamine in strain JC673^T^ and *G. obscuriglobus* UQM 2246^T^ is spermidine. However, in strain JC673^T^, putrescine is also present. Additional unidentified polyamines are present in all strains (Figure S4). MK-6 is the predominant quinone in all three strains.

### 2.6. Ethyl Acetate Extracts of the Novel Isolates Show Antimicrobial Activities

**Figure 6:**
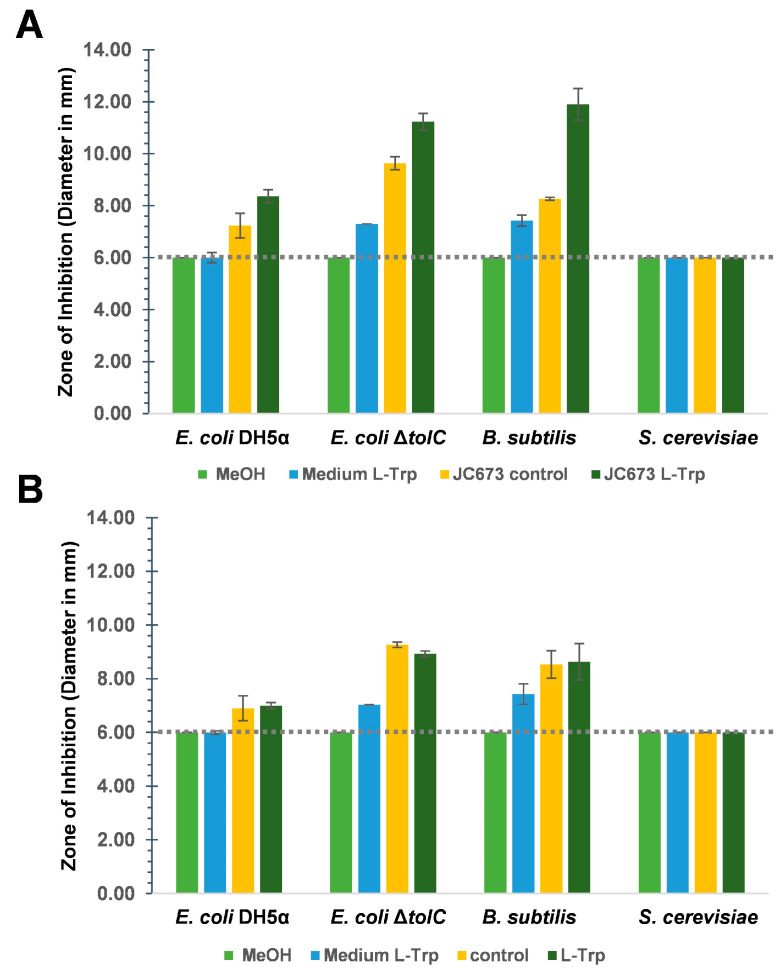
**Antimicrobial disc assay with extracts of cultures from (A) strain JC673^T^ and (B) *G. obscuriglobus* DSM 5831^T^**. The average value of measured zones of inhibition including the disc (6 mm, indicated by the dotted line) from three technical replicates are shown. The error bars indicate the standard deviation. MeOH: solvent negative control, medium l-Trp: ethyl acetate extracts of medium with 1 mM l-Trp (without cells), control: ethyl acetate extracts of cultures without l-Trp supplementation, and l-Trp: ethyl acetate extracts of cultures supplemented with 1 mM l-Trp.

Tests for antimicrobial activity were performed with strain JC673^T^ and *G. obscuriglobus* DSM 5831^T^ (same as UQM 2246^T^). Experiments were carried out using a previously described disk diffusion method (Figure 6 and Figure S5, Table S3) [7]. The measured diameter of growth inhibition zones also includes the diameter of the disk of 6 mm (=no activity). Growth inhibition caused by extracts of JC673^T^ and *G. obscuriglobus* cultures was tested for both, Gram-positive (*Bacillus subtilis*) and Gram-negative (*Escherichia coli*) bacterial test cultures and for the yeast *Saccharomyces cerevisiae.* The supplementation of the aromatic amino acid l-tryptophan (l-Trp) as potential precursor for bioactive compound biosynthesis was tested in addition to the standard cultivation medium for the planctomycetal strains. Already the extracts of planctomycetal cultures in the standard cultivation medium showed antimicrobial activity against the bacterial test cultures but not against the yeast. The inhibitory effect was stronger against an *E. coli* Δ*tolC* strain compared to *E. coli* DH5α. The gene *tolC* encodes a membrane protein that is part of several efflux systems for toxic compounds. Hence, the deletion mutant is more susceptible to compounds that are otherwise efficiently exported by efflux pumps involving TolC.

Small inhibition zones of *E. coli* Δ*tolC* were obtained for extracts of medium controls supplemented with 1 mM l-Trp. This effect may be due to its hypersensitivity to an increased concentration of this aromatic amino acid. In line with this assumption, *E. coli* DH5α, and *B. subtilis* DSM10^T^ did not show any growth inhibition zone when extracts of the medium control supplemented with l-Trp were used (Figure 6A,B and Figure S5I–II, Table S3A,B). The observed inhibitory effect of extracts of both *Gemmata* strains was stronger against the Gram-positive *B. subtilis* than against the Gram-negative *E. coli* (Figure 6A,B and Figure S5I–III, Table S3A,B). Like for kanamycin, which served as a positive control for antibacterial activity, inhibitory effects of extracts of both strains were not observed against *S. cerevisiae* (Figure 6A,B and Figure S5I–IV, Table S3A,B). Remarkably, for strain JC673^T^, the ethyl acetate extract of a culture in medium supplemented with 1 mM l-Trp had a more pronounced inhibitory activity against both the Gram-positive (*B. subtilis*: 11.9 mm) and Gram-negative test strains (*E. coli* Δ*tolC*: 11.2 mm; *E. coli* DH5α:: 8.4 mm) compared to extracts from standard medium without additionally supplemented l-Trp (control, C) (*B. subtilis*: 8.3 mm; *E. coli* Δ*tolC*: 9.8 mm; *E. coli* DH5α:: 7.2 mm) (Figure 6A,B and Figure S5I–IV, Table S3A,B). A direct effect of the supplemented l-Trp was excluded since the growth of the test strains (except the Δ*tolC* mutant) was not affected by extracts of medium supplemented with the same amount of l-Trp. Strain JC673^T^ and *G. obscuriglobus* gave the same inhibition pattern in the obtained extracts, however, the activity was stronger for extracts derived from the novel isolate. Aqueous extracts of both strains did not show significant antimicrobial activity against any of the test strains. The supplementation with l-Trp did not alter growth behaviour or cell morphology of the tested strains over a cultivation time of four weeks (Figure S6A,B).

**Figure 7:**
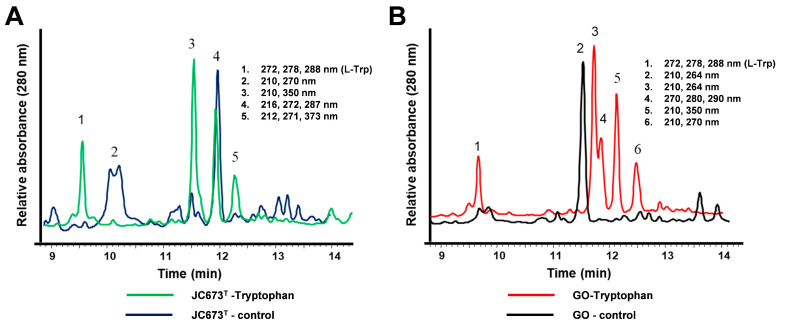
**HPLC analysis of culture extracts.** HPLC chromatograms of ethyl acetate extracts from cultures of (**A**) strain JC673^T^ and (**B**) *G. obscuriglobus* DSM 5831^T^. “Tryptophan” (green and red line, respectively): ethyl acetate extract from l-Trp supplemented cultures; “control” (blue and black line, respectively): ethyl acetate extract of culture in the same medium but without supplementation of l-Trp). Peaks in the chromatograms are numbered. For each of the peaks, the respective absorption maxima in the UV/Vis spectrum are provided. Same peak numbers in the chromatograms do not necessarily indicate the same compounds.

An HPLC analysis of ethyl acetate-extracted cultures yielded five to six peaks that were not present in the negative control (=culture without l-Trp supplementation) indicating that this amino acid might act as precursor for yet unidentified compounds (Figure 7). According to the annotated genome-encoded features of the two isolates, both lack the set of enzymes required for the catabolism of l-Trp via kynurenine. Although supplemented l-Trp is likely incorporated into nascent proteins during protein biosynthesis, the increased antimicrobial activity can probably not be attributed to the availability of l-Trp as an additional carbon source. This is also supported by the identification of residual L-Trp in the extracts during HPLC analysis. A functional catabolic pathway should have led to the complete consumption of L-Trp.

The bioactivity and the results of the chromatographic analysis support the production of compounds with antimicrobial activity by the tested strains. The cultivation of the novel isolates can be easily scaled up to get sufficient material for more detailed analyses. These include the isolation of the individual compounds by preparative HPLC analyses and fractionation. Elucidation of their chemical structure and testing the activity against a broader range of microbial species including pathogens will then be performed based on the fractionated extracts.

### 2.7. Description of Gemmata algarum *sp. nov.*

*Gemmata algarum* sp. nov. (al.ga’rum. L. fem. n. *alga*, seaweed, alga; L. pl. gen. n. *algarum*, of algae, referring to the isolation of the type strain from an algal mat).

Colonies of the species are pink-pigmented. Cells are spherical to ovoid, have a diameter of 1.4–2.2 µm and divide asymmetrically by budding. Cells are aerobic. NaCl is not required for growth but concentrations of up to 4% (*w*/*v*) NaCl are tolerated. Optimum pH and temperature for growth of the type strain are 8.0 (range 6.0–9.0) and 23–25 °C (range 10–30 °C), respectively. *N*-acetylglucosamine is not obligate for growth. d-Glucose, sucrose, mannose, xylose, ascorbate, starch, fructose, galactose, lactose, and maltose are utilized as carbon and energy source. Mannitol, propionate, inositol, malic acid, succinate, benzoic acid, fumarate, pyruvate, and sorbitol are not utilized. Ammonium sulphate, glycine, l-isoleucine, l-methionine, l-phenylalanine, l-proline, l-serine, peptone, yeast extract, sodium nitrate, l-glutamate, and l-glutamine can serve as nitrogen source for growth. Nitrogen sources like l-arginine, l-histidine, l-lysine, dl-threonine, l-cysteine, and l-tyrosine did not support growth. Nitrate is not reduced. The API ZYM kit showed positive signals for alkaline phosphatase, esterase (C4), esterase lipase (C8), leucine aryl amidase, α-chymotrypsin, naphthol-AS-BI-phosphohydrolase, and acid phosphatase. No activity was observed for lipase (C14), valine aryl amidase, cysteine aryl amidase, trypsin, α-galactosidase, β-glucuronidase, α-glucosidase, β- glucosidase, β-galactosidase, *N*-acetyl-β-glucosaminidase, α-mannosidase, and α-fucosidase. Major fatty acids are C_18:_0, C_16:1_ω5c, and C_16:0_. Minor fatty acids include C_14:0,_ *anteiso*-C_15:0,_ C_16:0_ *N*-alcohol, C_17:0_, *iso*-C_17:0_ 3-OH, C_18:3_ω6/9/12c, C_18:1_ω9c, C_18:1_ω5c, C_19:0,_ *iso*-C_19:0,_ C_20:0,_ C_17:1_ *iso* I/*anteiso* B, C_18:1_ω6c, and C_18:2_ ω6/9c/*anteiso*-C_18:0_. Polar lipids comprise phosphatidylcholine (PC), phosphatidylethanolamine (PE), several unidentified amino lipids, unidentified phospholipids, other unidentified lipids and an unidentified choline lipid. Spermidine and putrescine are the poly-amines produced. The genomic DNA G+C content is around 67.6%. The type strain is JC673^T^ (=KCTC 72851^T^ = NBRC 114340^T^). It was isolated from an algal mat from a wetland located close to the village Pallikkara in the south-western part of India (exact location: 12°25′15.8″ N 75°01′54.1″ E).

## 3. Conclusions and Outlook

Two new spherical and pink-pigmented members of the phylum *Planctomycetota* were isolated from algal mats in two different locations in India. Their polyphasic characterization suggests that both belong to a novel species of the genus *Gemmata*. Along with *G. obscuriglobus* DSM 5831^T^, extracts of strain JC673^T^ showed antibacterial activity. The activity was more pronounced when the aromatic amino acid l-Trp was supplemented to the cultures. The obtained results support the notion of the role of the phylum *Planctomycetota* as yet untapped source of bioactive compounds. Fractionation of the extracts and structure elucidation of the compound(s) responsible for the observed activity will be the starting point for more detailed analyses including cytotoxicity.

## 4. Materials and Methods

### 4.1. Habitat and Isolation

An algal mat was collected from the wetland located in Kerala in the south-western part of India, (village: Pallikkara, 12°25′15.8″ N 75°01′54.1″ E). Another algal mat was collected from the wall of the Central Marine Fisheries Research Institute (CMFRI) building in Mandapam (exact coordinates 09°16′29.3″ N 79°07′47.3″ E). The algal mat was growing under the steady flow of water leaking from a water tank placed on the roof. The CMFRI is situated in Tamil Nadu in the south-eastern part of India,. At the time of sample collection, the sampling spots had a pH of 7.0–7.5 and a temperature of 22 °C. Algal mat samples were subjected to enrichment and cultivation in a medium [20,24] containing (per litre distilled water; pH 7.0): *N*-acetylglucosamine, 2.0 g; KH_2_PO_4_, 0.1 g; peptone, 0.1 g; yeast extract, 0.1 g; vitamin solution, 10 mL; Hutner’s basal salts, 20 mL, prepared in distilled water. The antibiotics streptomycin, 400 mg/L, ampicillin, 200 mg/L and cycloheximide, 25 mg/L were added to the medium. The vitamin solution contained (in mg/L): vitamin B_12_, 0.2; biotin, 4; thiamine-HCl × 2 H_2_O, 10; calcium pantothenate, 10; folic acid, 4; riboflavin, 10; nicotinamide, 10; *p*-aminobenzoic acid, 10; pyridoxine HCl, 20. Hutner’s basal salts contained (in g/L): nitrilotriacetic acid, 10; MgSO_4_ × 7 H_2_O, 30; CaCl_2_ × 2 H_2_O, 3.5; (NH_4_)_6_MoO_7_O_24_ × 4 H_2_O, 0.01; FeSO_4_ × 7 H_2_O, 0.1; and metal stock solution, 50 mL. The metal stock solution contained (in g/L): Na-EDTA, 0.25; ZnSO_4_ × 7 H_2_O, 1.1; FeSO_4_ × 7 H_2_O, 0.5; MnSO_4_ × H_2_O, 0.15; CuSO_4_ × 5 H_2_O, 0.04; Co(NO_3_)_2_ × 6 H_2_O, 0.025; Na_2_B_4_O_7_ × 10 H_2_O, 0.018.

The samples (20 mg) were mixed with 10 mL medium in different serum vials of 50 mL capacity and sealed with butylated rubber stoppers. The serum vials were then incubated for fifteen days at 25 °C to enrich members of the phylum *Planctomycetota* by exploiting their natural resistance to the used antibiotics (cycloheximide was used to prevent fungal growth, streptomycin, and ampicillin were used to prevent growth of other bacteria). After fifteen days of incubation, a pink globular bacterial aggregate was observed at the bottom of two of the serum vials. The aggregates were streaked on agar plates containing the same medium. After two weeks of incubation, pink colonies appeared along with white colonies (appeared within 2 days) on the agar plates. The pink colonies were purified through repeated streaking. Pure cultures were maintained on agar plates by repeated sub-culturing and preserved at 4 °C. Purified cultures were grown in the above-mentioned medium without antibiotics, unless otherwise stated. The pink-coloured strains isolated from the algal mat of the wetland and the wall of the CMFRI building were designated JC673^T^ and JC717, respectively.

### 4.2. DNA Isolation, 16S rRNA Gene Sequencing and BLAST Analysis

The commercial Nucleo-pore gDNA Fungal Bacterial Mini Kit (Genetix Biotech Asia Pvt. Ltd., New Delhi, India) was used for genomic DNA isolation which was then used for 16S rRNA gene amplification and genome sequencing. For the 16S rRNA gene amplification, the *Planctomycetota*-specific primer F40 [29] and the universal primer R1388 [30] were used. For the PCR amplification, a TAKARA master mix (EmeraldAmp GT PCR Master Mix) was used. Thermocycler conditions were as follows: an initial denaturation step (94 °C for 10 min) followed by 33 cycles of denaturation (94 °C for 1 min), annealing (52 °C for 54 s), and extension (72 °C for 1.4 min). The amplified PCR products were sent to AgriGenomePvt. Ltd. (Kochi, India) for purification and 16S rRNA gene sequencing. The sequence information obtained was used for a BLAST search in the EzBioCloud database [31] and on the NCBI website [32].

### 4.3. Genome Sequencing and In-Silico Analysis of Genome-Encoded Features

Whole-genome sequencing (WGS) of strains JC673^T^ and JC717 was outsourced to AgriGenome Pvt. Ltd., Kochi, India. WGS was carried out using an Illumina NovaSeq6000 platform and paired-end libraries were generated with a sequence coverage of at least 100×. The Unicycler v. 0.4.8 assembly software [33] was used for de novo assembly with default k-mer sizes and for all further downstream analyses. The ContEst service [31] was used for the detection of any possible contamination. The RAST server [34] and the NCBI Prokaryotic Genome Annotation Pipeline (PGAP) [35] were used for open reading frame calling and annotation. The pangenome analysis was performed with the pangenomics workflow of anvi’o v. 7.1 [36]. The genome-wide analysis of orthologous protein clusters was carried out using the OrthoVenn2 tool (https://orthovenn2.bioinfotoolkits.net/ (accessed on 20 October 2023) [37] with the default threshold E-value (10^−5^). In silico metabolic characterization of strains JC673^T^ and JC717 was carried out based on the genome information using KEGG mapper [38]. The ‘Search with HMMs of Environmental Bio element families-v1’ tool in Kbase was used to identify genes coding for enzymes involved in different environmental bioelement cycles using HMMs [39]. Biosynthetic gene clusters putatively involved in the biosynthesis of secondary metabolites were predicted using antiSMASH 7.0 with relaxed strictness and all extra features including beta features activated [40].

### 4.4. Phylogenetic Analysis

The 16S rRNA gene sequences of strains JC673^T^ and JC717 were extracted from the respective genomes using ContEst16S (https://www.ezbiocloud.net/tools/contest16s, accessed on 15 October 2023) and the analysis of identity was performed using NCBI BLAST [32]. The maximum-likelihood 16S rRNA gene sequence-based phylogeny was computed for the novel strains and the described type strains of species in the current phylum *Planctomycetota* (as of October 2023). The alignment of the 16S rRNA gene sequences was performed with ClustalW [41] and FastTree was used for tree reconstruction with 1000 bootstrap replications [42]. Three 16S rRNA gene sequences from strains outside of the phylum *Planctomycetota* but part of the *Planctomycetota*-*Verrucomicrobiota*-*Chlamydiota* (PVC) superphylum, namely *Opitutus terrae* (NCBI acc. no. AJ229235), *Kiritimatiella glycovorans* (acc. no. NR_146840) and *Lentisphaera araneosa* (acc. no. NR_027571), served as outgroup. The MLSA-based phylogenetic analysis was performed using autoMLST with 500 bootstrap replicates [43]. The analysis was conducted with the “autoMLST-simplified-wrapper” script available on GitHub (https://github.com/KatSteinke/automlst-simplified-wrapper, accessed on 15 October 2023) and was based on at least 30 single-copy protein-coding genes. The analysis included all reference genomes of strains belonging to the current order *Gemmatales*. The genomes of *Rhodopirellula baltica* SH1^T^ (GenBank acc. no. BX119912.1), *Pirellula staleyi* DSM 6068^T^ (acc. no. CP001848.1) and *Blastopirellula marina* DSM 3645^T^ (acc. no. GCA_000153105.1) (all belonging to the order *Pirellulales*) served as outgroup. Phylogenetic trees were visualized with iTOL v6 [44]. The 16S rRNA gene similarity matrix was obtained with TaxonDC [45] based on the ClustalW alignment that was also used for the construction of the phylogenetic tree. The AAI and ANI values were calculated using respective scripts of the enveomics collection [46]. The digital DNA-DNA hybridization (dDDH) analysis was performed using the Genome-to-Genome Distance Calculator 3.0 of the DSMZ (https://ggdc.dsmz.de/ggdc.php) (accessed on 15 October 2023).

### 4.5. Physiological Analyses

For organic substrate and nitrogen source utilization, a basal medium was used as previously described [47] with slight modifications. The medium was supplemented with a small amount of yeast extract (0.05% *w*/*v*). For organic substrate utilization (NH_4_)_2_SO_4_ (0.1% *w*/*v*) was used as nitrogen source and growth was tested with different organic substrates at a concentration of 0.1% (*w*/*v*). For nitrogen source utilization, glucose (0.1% *w*/*v*) was used as organic carbon source and growth was tested with different nitrogen substrates at a concentration of 0.1% (*w*/*v*). Ten millilitres of broth in test tubes (25 × 250 mm) were used for determining the utilization of organic carbon/nitrogen sources [48]. The NaCl tolerance (final concentration of 1–10% *w*/*v*, at an interval of 1% *w*/*v*) was tested at 25 °C and pH 8.0. The optimal temperature for growth was determined in modified M30 medium [22] at pH 8.0 (tested temperatures: 5, 10, 15, 20, 25, 30, 35, 40 °C). The pH range and optimum (4.0, 5.0, 6.0, 7.0, 8.0, 9.0, 10.0) for growth were tested at 25 °C in buffered medium as previously described [49]. Enzyme activities were assayed using the API ZYM kit (Biomerieux, Marcy-l’Étoile, France) based on the manufacturer’s protocol.

### 4.6. Chemotaxonomic Characterization

For fatty acid analysis, exponentially growing cells were harvested by centrifugation (10,000× *g* for 15 min at 4 °C) at a cell density of 70% of the maximum optical density (100% = OD_660_ of 0.9). Cellular fatty acids were methylated, separated, and identified according to the instructions for the Microbial Identification System [Microbial ID; MIDI 6.0 version; method RTSBA6] [50], which was carried out by Royal Research Labs, Secunderabad, India. Polar lipids were extracted, separated, and characterized as described [51,52]. Quinones were extracted with a chloroform/methanol (2:1, *v*/*v*) mixture, purified by TLC and analysed by HPLC [53]. Polyamines were extracted and identified according to a published method [21].

### 4.7. Microscopy and Image Analysis

Cell morphological features like size, shape, and mode of cell division were investigated using light microscopy. For image acquisition, a Nikon Eclipse Ti2 was used, equipped with a Nikon DS-Ri2 camera with a Nikon DS-F2.5 camera adapter, a Plan Apo λ 100× objective (with phase ring for phase contrast images) and a Plan Apo λ 100× objective (without phase ring for differential interference contrast images). The Eclipse Ti2 was used with NIS Elements Version 5.42.03, which was also used for image stitching (0% overlap). Images were converted from native file format into jpeg and tiff formats using FIJI Version 2.9.0 [54]. The first one was used for visualisation only; a scale bar was added, and brightness and contrast were adapted. The tiff images were fed into the image analysis pipeline consisting of semi-automated cell size determination with BacStalk Version 1.8 [55] and data visualization with SuperPlotsOfData [56]. In total, 497 cells of one biological replicate were analysed and proper cell selection by the software was checked manually for every cell.

Cell morphological features were also observed using field emission scanning electron microscopy (FESEM) or Transmission electron microscopy (TEM). For FESEM, 1 mL of a log phase culture was centrifuged at 7000× *g* for 10 min at 4 °C. The resulting cell pellet was washed by re-suspending in sterile MilliQ water and centrifuged at 7000× *g* for 10 min at 4 °C. The pellet or cells were fixed in 2.5% (*v*/*v*) glutaraldehyde solution and incubated for six hours at 4 °C. Cells were dehydrated sequentially with an increased ethanol concentration from 10–100% (*v*/*v*) (in a 10% interval). At last, the cells were resuspended in 100% ethanol. Ten millilitres of sample were kept on a small size glass slide, which was placed on the stab with adhesive tape [23]. Finally, stabs were kept for gold sputtering for six minutes and then cell morphology and division were observed under the FESEM (Philips XL3O) in the facility of the School of Physics, University of Hyderabad. For TEM, ultrathin sectioning of log phase cells was outsourced to RUSKA Diagnostic, Hyderabad. Prior to ultrathin sectioning, cells were fixed in 3% (*v*/*v*) glutaraldehyde in 0.1 M phosphate buffer (pH 7.2) for 24 h at 4 °C and post-fixed in 2% (*w*/*v*) aqueous osmium tetroxide in the same buffer for 2 h and dehydrated in a graded series of ethanol, infiltrated, and embedded in araldite 6005 resin or spur resin. Ultra-thin (50–70 nm) sections were made with a glass knife on ultra-microtome (Leica Ultra cut UCT-GA-D/E-100), mounted on copper grids and stained with saturated aqueous uranyl acetate and counter stained with Reynolds lead citrate. The sections of the cells were mounted on copper grids and observed under the TEM (H-7500 Hitachi) in the Centre for Cellular & Molecular Biology (CCMB), Hyderabad.

### 4.8. Metabolite Extraction and Antimicrobial Screening

Single colonies of strain JC673^T^ and *G. obscuriglobus* DSM 5831^T^ were inoculated in four conical flasks (500 mL, Borosil) (each strain in two different flasks) containing 200 mL of DSMZ medium 629 and incubated at 25 °C on a rotary shaker (100 rpm). At the mid-exponential phase (on day 4), one of the flasks of each culture was supplemented with 1 mM sterile l-Trp followed by further incubation for 6–7 days. The culture without supplemented l-Trp served as control. At the stationary phase (after 10 days of incubation and at an OD_600_ of 1.1 and 1.3 for strain JC673^T^ and *G. obscuriglobus* DSM 5831^T^, respectively) cultures were harvested by centrifugation (4500 rpm at 4 °C, 10 min). The culture supernatants were acidified to pH 2.0 by the addition of 5 M HCl. Culture supernatants were subsequently extracted twice using two-phase extraction with ethyl acetate (1:1, *v*/*v*) as solvent and were pooled afterwards. During the extraction procedure, also the polar (aqueous) phase was collected in separate flasks. The ethyl acetate-extracted pool was dried under vacuum using a rotary evaporator (Heidolph, Germany) at 35 °C. The dried extract was dissolved in 0.5 mL HPLC-grade methanol and filtered through a 0.22 µm membrane (Icon Pall). The aqueous phase was also dried using a rotary evaporator (Heidolph, Germany) at 40 °C followed by dissolving in 1 mL methanol. The extracts were then tested for antimicrobial activity by analysing for zones of inhibition on agar plates. For this purpose, 20 µL of each extract was pipetted on three sterile discs and after 5 min each disc was incubated on different agar plates on which one of the following strains has been freshly streaked: *E. coli* DH5α, *E. coli* Δ*tolC*, *S. cerevisiae*, and *B. subtilis* [7]. The experiment was performed in technical triplicates. The protocol for the HPLC analysis was adapted from a previously published study [57]. A linear gradient program was employed to separate the metabolites. Water with 1% (*v*/*v*) acetic acid (solvent A) and acetonitrile (solvent B) (HPLC-grade) were used as mobile phase with a flow rate of 1.5 mL/min. The absorption spectra were recorded with a photodiode.

## Figures and Tables

**Table 1:** Differences in the characteristics of strains JC673^T^, JC717, *Gemmata obscuriglobus* UQM 2246^T^, *G. massiliana* IIL30^T^, and *G. palustris* G18^T^.

Characteristics	JC673^T^	*G. obscuriglobus* UQM 2246^T^	*G. massiliana* IIL30^T^ (#)	*G. palustris* G18^T^ (#)
Cell size (µm)	1.4–2.2	1.4–3.0	1.1–2.1	1.5–2.8
pH range (optimum)	6.0–9.0 (8.0)	7.0–9.0 (7.0)	6.0–8.0 (8.0)	6.0–8.0 (7.0)
NaCl range (% (*w*/*v*))	0–4 (2)	0–1 (0)	0–0.6 (ND)	0–1.25 (ND)
Temperature range (optimum)	10–30 (23–25)	16–35 (26–28)	25–37 (30)	4–28 (15–20)
**Nitrogen source utilization**				
l-Isoleucine	+	−	ND	ND
l-Methionine	+	−	ND	ND
l-Phenylalanine	+	−	ND	ND
l-Proline	+	−	ND	ND
dl-Threonine	−	+	ND	ND
l-Histidine	−	+	ND	ND
**Carbon source utilization**				
Ascorbate	+	−	ND	ND
Starch	+	−	ND	−
Fructose	+	−	ND	ND
Galactose	+	−	ND	ND
Maltose	−	+	ND	ND
Fumarate	−	+	ND	ND
**Nitrate Reduction**	+	−	ND	−
**Antibiotic resistance**				
Ampicillin	R	R	ND	S
Streptomycin	R	R	ND	S
**Activity of enzymes**				
Valine arylamidase	−	+	+	+
β-Galactosidase	−	+	−	−
α-Chymotrypsin	+	−	−	+
α-Glucosidase	-	+	−	−
β-Glucosidase	-	+	−	−
Cysteine arylamidase	−	−	−	+
Trypsin	−	−	+	+
**Fatty acid composition**				
*iso*-C_19:0_	+	−	ND	ND
C_18:0_ ω6,9c/ante-C_18:0_	+	−	ND	ND
*iso*-C_17:0_ 3-OH	−	−	ND	ND
**Major Polar lipids**				
Phosphatidylcholine	−	−	+	+
Phosphatidylethanolamine	−	−	−	+
**Major Polyamines**				
Putrescine	+	−	ND	ND
**Genomic features**				
DNA G+C content (mol%)	67.6	67.4	64.0	65.0
Genome size (Mb)	8.20	9.03	10.14	9.23
Number of genes	6505	7408	8383	7539
RNAs	88	106	102	97
CRISPRs	6	3	4	3

Data for strain JC673^T^ and *Gemmata obscuriglobus* UQM 2246^T^ was analysed in this study. #, data taken from the species description manuscripts [9,19]. ND, Not determined.; R, resistant; and S, sensitive; +, positive signal/substrate utilized for growth; −, negative signal, substrate not utilized for growth.

## Data Availability

The GenBank/EMBL/DDBJ accession numbers for the 16S rRNA gene sequences of strains JC673^T^ and JC717 are OR644607 and OR644608, respectively. The GenBank/EMBL/DDBJ accession numbers for the whole genome shotgun sequence for strains JC673^T^ and JC717 are JAXBLV000000000 and JAXBLW000000000, respectively.

## Supplementary Materials

The following supporting information can be downloaded at: https://www.mdpi.com/article/10.3390/md22010010/s1, Table S1: Comparison of genomic features of the two novel isolates; Table S2: Analysis of fatty acid composition; Table S3: Zones of inhibition caused by culture extracts of the novel isolates; Figure S1: Clusters of orthologous proteins; Figure S2: Numbers of biosynthetic gene clusters predicted by antiSMASH; Figure S3: Analysis of polar lipids; Figure S4. Analysis of polyamines; Figure S5: Agar disc diffusion assays for testing antimicrobial activity; Figure S6: Phenotypical analysis of stationary cultures.

## Abbreviations

NCBI, National Centre for Biotechnology Information; ANI, Average Nucleotide Identity; AAI, Average Amino Acid Identity; dDDH, digital DNA-DNA Hybridization; HPLC, High Performance Liquid Chromatography; TLC, Thin Layer Chromatography; SEM, Scanning Electron Microscopy; TEM, Transmission Electron Microscopy; KCTC, Korean Collection for Type Cultures; NBRC, Biological Resource Centre, National Institute of Technology and Evaluation (NITE), Japan; DSMZ, Deutsche Sammlung von Mikroorganismen und Zellkulturen; HMM, Hidden Markov Model.

## References

[1] Van Teeseling M.C.F., Jogler C. (2021). Cultivation of elusive microbes unearthed exciting biology. Nat. Commun..

[2] Jiao J.-Y., Liu L., Hua Z.-S., Fang B.-Z., Zhou E.-M., Salam N., Hedlund B.P., Li W.-J. (2020). Microbial dark matter coming to light: Challenges and opportunities. Natl. Sci. Rev..

[3] Boedeker C., Schüler M., Reintjes G., Jeske O., van Teeseling M.C.F., Jogler M., Rast P., Borchert D., Devos D.P., Kucklick M. (2017). Determining the bacterial cell biology of Planctomycetes. Nat. Commun..

[4] Rivas-Marin E., Peeters S.H., Claret Fernández L., Jogler C., van Niftrik L., Wiegand S., Devos D.P. (2020). Non-essentiality of canonical cell division genes in the planctomycete *Planctopirus limnophila*. Sci. Rep..

[5] Wiegand S., Jogler M., Boedeker C., Pinto D., Vollmers J., Rivas-Marín E., Kohn T., Peeters S.H., Heuer A., Rast P. (2020). Cultivation and functional characterization of 79 planctomycetes uncovers their unique biology. Nat. Microbiol..

[6] Kallscheuer N., Jogler C. (2021). The bacterial phylum *Planctomycetes* as novel source for bioactive small molecules. Biotechnol. Adv..

[7] Belova S.E., Saltykova V.A., Dedysh S.N. (2020). Antimicrobial Activity of a Novel Freshwater Planctomycete *Lacipirellula parvula* PX69^T^. Microbiology.

[8] Vitorino I.R., Lage O.M. (2022). The *Planctomycetia*: An overview of the currently largest class within the phylum Planctomycetes. Antonie Van Leeuwenhoek.

[9] Ivanova A.A., Kulichevskaya I.S., Dedysh S.N. (2021). *Gemmata palustris* sp. nov., a Novel Planctomycete from a Fen in Northwestern Russia. Microbiology.

[10] Kulichevskaya I., Serkebaeva Y., Kim Y., Rijpstra I., Sinninghe Damste J., Liesack W., Dedysh S. (2012). *Telmatocola sphagniphila* gen. nov., sp. nov., a Novel Dendriform Planctomycete from Northern Wetlands. Front. Microbiol..

[11] Kulichevskaya I.S., Baulina O.I., Bodelier P.L.E., Rijpstra W.I.C., Damsté J.S.S., Dedysh S.N. (2009). *Zavarzinella formosa* gen. nov., sp. nov., a novel stalked, *Gemmata*-like planctomycete from a Siberian peat bog. Int. J. Syst. Evol. Microbiol..

[12] Kulichevskaya I.S., Ivanova A.A., Baulina O.I., Rijpstra W.I.C., Sinninghe Damsté J.S., Dedysh S.N. (2017). *Fimbriiglobus ruber* gen. nov., sp. nov., a *Gemmata*-like planctomycete from Sphagnum peat bog and the proposal of *Gemmataceae* fam. nov. Int. J. Syst. Evol. Microbiol..

[13] Kulichevskaya I.S., Ivanova A.A., Naumoff D.G., Beletsky A.V., Rijpstra W.I.C., Sinninghe Damsté J.S., Mardanov A.V., Ravin N.V., Dedysh S.N. (2020). *Frigoriglobus tundricola* gen. nov., sp. nov., a psychrotolerant cellulolytic planctomycete of the family *Gemmataceae* from a littoral tundra wetland. Syst. Appl. Microbiol..

[14] Kulichevskaya I.S., Naumoff D.G., Miroshnikov K.K., Ivanova A.A., Philippov D.A., Hakobyan A., Rijpstra W.I.C., Damsté J.S.S., Liesack W., Dedysh S.N. (2020). *Limnoglobus roseus* gen. nov., sp. nov., a novel freshwater planctomycete with a giant genome from the family *Gemmataceae*. Int. J. Syst. Evol. Microbiol..

[15] Kallscheuer N., Rast P., Jogler M., Wiegand S., Kohn T., Boedeker C., Jeske O., Heuer A., Quast C., Glöckner F.O. (2021). Analysis of bacterial communities in a municipal duck pond during a phytoplankton bloom and isolation of *Anatilimnocola aggregata* gen. nov., sp. nov., *Lacipirellula limnantheis* sp. nov. and *Urbifossiella limnaea* gen. nov., sp. nov. belonging to the phylum *Planctomycetes*. Environ. Microbiol..

[16] Franzmann P.D., Skerman V.B.D. (1984). *Gemmata obscuriglobus*, a new genus and species of the budding bacteria. Antonie Van Leeuwenhoek.

[17] Seeger C., Butler M.K., Yee B., Mahajan M., Fuerst J.A., Andersson S.G.E. (2017). *Tuwongella immobilis* gen. nov., sp. nov., a novel non-motile bacterium within the phylum Planctomycetes. Int. J. Syst. Evol. Microbiol..

[18] Elcheninov A.G., Podosokorskaya O.A., Kovaleva O.L., Novikov A.A., Toshchakov S.V., Bonch-Osmolovskaya E.A., Kublanov I.V. (2021). *Thermogemmata fonticola* gen. nov., sp. nov., the first thermophilic planctomycete of the order *Gemmatales* from a Kamchatka hot spring. Syst. Appl. Microbiol..

[19] Aghnatios R., Cayrou C., Garibal M., Robert C., Azza S., Raoult D., Drancourt M. (2015). Draft genome of *Gemmata massiliana* sp. nov, a water-borne *Planctomycetes* species exhibiting two variants. Stand. Genom. Sci..

[20] Lhingjakim K.L., Smita N., Kumar G., Jagadeeshwari U., Ahamad S., Sasikala C., Ramana C.V. (2022). *Paludisphaera rhizosphaereae* sp. nov., a new member of the family *Isosphaeraceae*, isolated from the rhizosphere soil of *Erianthus ravennae*. Antonie Van Leeuwenhoek.

[21] Kumar D., Gaurav K., Pk S., A S., Uppada J., Ch S., Ch V.R. (2020). *Gimesia chilikensis* sp. nov., a haloalkali-tolerant planctomycete isolated from Chilika lagoon and emended description of the genus *Gimesia*. Int. J. Syst. Evol. Microbiol..

[22] Kumar G., Jagadeeshwari U., Sreya P., Shabbir A., Sasikala C., Ramana C.V. (2022). A genomic overview including polyphasic taxonomy of *Thalassoroseus pseudoceratinae* gen. nov., sp. nov. isolated from a marine sponge, *Pseudoceratina* sp.. Antonie Van Leeuwenhoek.

[23] Kumar G., Kumar D., Jagadeeshwari U., Sreya P.K., Shabbir A., Sasikala C., Ramana C.V. (2021). *Crateriforma spongiae* sp. nov., isolated from a marine sponge and emended description of the genus “*Crateriforma*”. Antonie Van Leeuwenhoek.

[24] Kumar G., Lhingjakim K.L., Uppada J., Ahamad S., Kumar D., Kashif G.M., Sasikala C., Ramana C.V. (2021). *Aquisphaera insulae* sp. nov., a new member in the family *Isosphaeraceae*, isolated from the floating island of Loktak lake and emended description of the genus *Aquisphaera*. Antonie Van Leeuwenhoek.

[25] Chun J., Oren A., Ventosa A., Christensen H., Arahal D.R., da Costa M.S., Rooney A.P., Yi H., Xu X.-W., De Meyer S. (2018). Proposed minimal standards for the use of genome data for the taxonomy of prokaryotes. Int. J. Syst. Evol. Microbiol..

[26] Meier-Kolthoff J.P., Klenk H.-P., Göker M. (2014). Taxonomic use of DNA G+C content and DNA–DNA hybridization in the genomic age. Int. J. Syst. Evol. Microbiol..

[27] Qin Q.-L., Xie B.-B., Zhang X.-Y., Chen X.-L., Zhou B.-C., Zhou J., Oren A., Zhang Y.-Z. (2014). A proposed genus boundary for the prokaryotes based on genomic insights. J. Bacteriol..

[28] Luo C., Rodriguez-R L.M., Konstantinidis K.T. (2014). MyTaxa: An advanced taxonomic classifier for genomic and metagenomic sequences. Nucleic Acids Res..

[29] Köhler T., Stingl U., Meuser K., Brune A. (2008). Novel lineages of Planctomycetes densely colonize the alkaline gut of soil-feeding termites (*Cubitermes* spp.). Environ. Microbiol..

[30] Stackebrandt E., Liesack W., Goebel B.M. (1993). Bacterial diversity in a soil sample from a subtropical Australian environment as determined by 16S rDNA analysis. FASEB J..

[31] Yoon S.H., Ha S.M., Kwon S., Lim J., Kim Y., Seo H., Chun J. (2017). Introducing EzBioCloud: A taxonomically united database of 16S rRNA gene sequences and whole-genome assemblies. Int. J. Syst. Evol. Microbiol..

[32] Johnson M., Zaretskaya I., Raytselis Y., Merezhuk Y., McGinnis S., Madden T.L. (2008). NCBI BLAST: A better web interface. Nucleic Acids Res..

[33] Wick R.R., Judd L.M., Gorrie C.L., Holt K.E. (2017). Unicycler: Resolving bacterial genome assemblies from short and long sequencing reads. PLOS Comput. Biol..

[34] Aziz R.K., Bartels D., Best A.A., DeJongh M., Disz T., Edwards R.A., Formsma K., Gerdes S., Glass E.M., Kubal M. (2008). The RAST Server: Rapid Annotations using Subsystems Technology. BMC Genom..

[35] Li W., O’Neill K.R., Haft D.H., DiCuccio M., Chetvernin V., Badretdin A., Coulouris G., Chitsaz F., Derbyshire M.K., Durkin A.S. (2020). RefSeq: Expanding the Prokaryotic Genome Annotation Pipeline reach with protein family model curation. Nucleic Acids Res..

[36] Eren A.M., Kiefl E., Shaiber A., Veseli I., Miller S.E., Schechter M.S., Fink I., Pan J.N., Yousef M., Fogarty E.C. (2021). Community-led, integrated, reproducible multi-omics with anvi’o. Nat. Microbiol..

[37] Xu L., Dong Z., Fang L., Luo Y., Wei Z., Guo H., Zhang G., Gu Y.Q., Coleman-Derr D., Xia Q. (2019). OrthoVenn2: A web server for whole-genome comparison and annotation of orthologous clusters across multiple species. Nucleic Acids Res..

[38] Kanehisa M., Sato Y. (2020). KEGG Mapper for inferring cellular functions from protein sequences. Protein Sci..

[39] Arkin A.P., Cottingham R.W., Henry C.S., Harris N.L., Stevens R.L., Maslov S., Dehal P., Ware D., Perez F., Canon S. (2018). KBase: The United States Department of Energy Systems Biology Knowledgebase. Nat. Biotechnol..

[40] Blin K., Shaw S., Kloosterman A.M., Charlop-Powers Z., van Wezel G.P., Medema M.H., Weber T. (2021). antiSMASH 6.0: Improving cluster detection and comparison capabilities. Nucleic Acids Res..

[41] Thompson J.D., Gibson T.J., Higgins D.G. (2003). Multiple Sequence Alignment Using ClustalW and ClustalX. Curr. Protoc. Bioinform..

[42] Price M.N., Dehal P.S., Arkin A.P. (2009). FastTree: Computing Large Minimum Evolution Trees with Profiles instead of a Distance Matrix. Mol. Biol. Evol..

[43] Alanjary M., Steinke K., Ziemert N. (2019). AutoMLST: An automated web server for generating multi-locus species trees highlighting natural product potential. Nucleic Acids Res..

[44] Letunic I., Bork P. (2021). Interactive Tree of Life (iTOL) v5: An online tool for phylogenetic tree display and annotation. Nucleic Acids Res..

[45] Tarlachkov S., Starodumova I. (2017). TaxonDC: Calculating the similarity value of the 16S rRNA gene sequences of prokaryotes or its regions of fungi. J. Bioinform. Genom..

[46] Rodriguez-R L.M., Konstantinidis K.T. (2016). The enveomics collection: A toolbox for specialized analyses of microbial genomes and metagenomes. PeerJ.

[47] Bondoso J., Albuquerque L., Nobre M.F., Lobo-da-Cunha A., da Costa M.S., Lage O.M. (2011). *Aquisphaera giovannonii* gen. nov., sp. nov., a planctomycete isolated from a freshwater aquarium. Int. J. Syst. Evol. Microbiol..

[48] Kaushik R., Sharma M., Gaurav K., Jagadeeshwari U., Shabbir A., Sasikala C., Ramana C.V., Pandit M.K. (2020). *Paludisphaera soli* sp. nov., a new member of the family *Isosphaeraceae* isolated from high altitude soil in the Western Himalaya. Antonie Van Leeuwenhoek.

[49] Bondoso J., Albuquerque L., Nobre M.F., Lobo-da-Cunha A., da Costa M.S., Lage O.M. (2015). *Roseimaritima ulvae* gen. nov., sp. nov. and *Rubripirellula obstinata* gen. nov., sp. nov. two novel planctomycetes isolated from the epiphytic community of macroalgae. Syst. Appl. Microbiol..

[50] Sasser M. (1990). Identification of Bacteria by Gas Chromatography of Cellular Fatty Acids.

[51] Kates M. (1972). Isolation, analysis and identification of lipids.

[52] Oren A., Duker S., Ritter S. (1996). The polar lipid composition of Walsby’s square bacterium. FEMS Microbiol. Lett..

[53] Imhoff J.F. (1984). Quinones of phototrophic purple bacteria. FEMS Microbiol. Lett..

[54] Schindelin J., Arganda-Carreras I., Frise E., Kaynig V., Longair M., Pietzsch T., Preibisch S., Rueden C., Saalfeld S., Schmid B. (2012). Fiji: An open-source platform for biological-image analysis. Nat. Methods.

[55] Hartmann R., van Teeseling M.C.F., Thanbichler M., Drescher K. (2020). BacStalk: A comprehensive and interactive image analysis software tool for bacterial cell biology. Mol. Microbiol..

[56] Goedhart J. (2021). SuperPlotsOfData—A web app for the transparent display and quantitative comparison of continuous data from different conditions. Mol. Biol. Cell.

[57] Mujahid M., Sasikala C., Ramana C.V. (2011). Production of indole-3-acetic acid and related indole derivatives from L-tryptophan by *Rubrivivax benzoatilyticus* JA2. Appl. Microbiol. Biotechnol..

