# Supplementary material for: Gemmata algarum, a Novel Planctomycete Isolated from an Algal Mat, Displays Antimicrobial Activity
Source: Mar Drugs. 2023 Dec 21;22(1):10. doi: 10.3390/md22010010 (PMC10817699; doi:10.3390/md22010010)
Supplement: Supplementary file 1 [file marinedrugs-22-00010-s001.zip › marinedrugs-2622906-supplementary.pdf]

Supplementary Materials

Gemmata algarum, a novel planctomycete isolated from an algal mat, displays antimicrobial activity

Gaurav Kumar[1,2,3,#], Nicolai Kallscheuer[2,#,] Mohammad Kashif[1], Shabbir Ahamad[1], Uppada Jagadeeshwari[4], Sreya Pannikurungottu[1], Tom Haufschild[2], Moses Kabuu[2], Chintalapati Sasikala[4], Christian Jogler[2,3,*] and Chintalapati Venkata Ramana[1,*]

[1] Department of Plant Sciences, School of Life Sciences, University of Hyderabad, P.O. Central University, Hyderabad 500046, India.
[2] Department of Microbial Interactions, Institute of Microbiology, Friedrich Schiller University, Jena, 07743, Germany.
[3] Cluster of Excellence Balance of the Microverse, Friedrich Schiller University, Jena, Germany.
[4] Bacterial Discovery Laboratory, Centre for Environment, Institute of Science and Technology, JNT University Hyderabad, Kukatpally, Hyderabad-500085, India.

[#] Both authors contributed equally and are considered as equal first authors
* Corresponding authors: Christian Jogler, christian.jogler@uni-jena.de; Chintalapati Venkata Ramana, cvr449@gmail.com

Supplementary Tables

Table S1. Comparison of genomic features of the two novel isolates.

Feature	JC673^T	JC717
G+C content (%)	67.6	67.6
Genome size (bp)	8,204,877	8,254,380
Number of contigs	253	303
Longest contig (bp)	311,477	176,178
N50 (bp)	94,033	68,100
Coding density (%)	85.0	84.9
Genes (total)	6,505	6,668
Protein-coding genes	6,364	6,542
Hypothetical proteins	2,013	2,095
Hypothetical proteins (%)	31.6	32.0
rRNA genes (5S-16S-23S)	1-1-1	2-1-1
tRNAs	81	87
CRISPR arrays	6	3
Completeness (%)	96.26	96.26
Contamination (%)	3.52	4.49

Table S2. **Analysis of fatty acid composition (in %).**

**Fatty acid**	**JC673^T**	***G. obscuriglobus* UQM 2246^T**
$C_{14:0}$	1.0	0.7
anteiso-$C_{15:0}$	0.4	-
$C_{16:0}$	7.1	5.9
$C_{16:1}$ ω5c	19.2	23.1
$C_{16:0}$ *N*-alcohol	0.6	-
$C_{17:0}$	0.8	0.8
iso-$C_{17:0}$ 3-OH	0.7	-
$C_{18:3}$ ω6,9,12c	0.5	0.8
$C_{18:0}$	52.4	54.2
$C_{18:1}$ω9c	1.0	1.8
$C_{18:1}$ω5c	1.5	1.8
$C_{19:0}$	3.0	2.9
iso-$C_{19:0}$	3.2	-
$C_{20:0}$	1.3	1.4
$C_{17:1}$ *iso* I/*anteiso* B	3.3	2.8
$C_{18:1}$ω6c/7c	3.3	3.8
$C_{18:2}$ ω6,9c/*anteiso*-$C_{18:0}$	0.5	-

Table S3. Zones of inhibition caused by culture extracts of the novel isolates. The table summarizes the measured diameters of the obtained zones of inhibition in mm including the disc (6 mm). R1-3: Replicates 1-3, SD: Standard deviation, MeOH: methanol, Trp: ethyl acetate extracts of L-Trp-supplemented cultures, Control: ethyl acetate extracts of cultures without L-Trp-supplementation., G.O, *G. obscuriglobus* UQM 2246^{T}. The diameter of the disk is 6.0 mm

.

Part A. Antimicrobial activity of *G. obscuriglobus* UQM 2246^{T} against microbial test cultures																				
	E. coli* Δ*tolC					***E. coli* DH5α**					***B. subtilis***					***S. cerevisiae***				
	**R1**	**R2**	**R3**	**Average**	**S.D**	**R1**	**R2**	**R3**	**Average**	**S.D**	**R1**	**R2**	**R3**	**Average**	**S.D**	**R1**	**R2**	**R3**	**Average**	**S.D**
**Me-OH**	6.0	6.0	6.0	**6.00**	0.00	6.0	6.0	6.0	**6.00**	0.00	6.0	6.0	6.0	**6.00**	0.00	6.0	6.0	6.0	**6.00**	0
**Medium + Trp**	7.0	7.1	7.0	**7.03**	0.06	6.0	6.0	6.0	**6.00**	0.00	7.7	7.6	7.0	**7.43**	0.38	6.0	6.0	6.0	**6.00**	0
**G.O_ Control**	9.0	9.8	9.0	**9.27**	0.46	6.9	6.8	7.0	**6.90**	0.10	9.1	8.1	8.4	**8.53**	0.51	6.0	6.0	6.0	**6.00**	0
**G.O_ Trp**	9.0	8.8	9.0	**8.93**	0.12	7.0	6.9	7.1	**7.00**	0.10	9.4	8.4	8.1	**8.63**	0.68	6.0	6.0	6.0	**6.00**	0

Part B. Antimicrobial activity of the strain JC673^{T} against microbial test cultures																				
	E. coli* Δ*tolC					***E. coli* DH5α**					***B. subtilis***					***S. cerevisiae***				
	**R1**	**R2**	**R3**	**Average**	**S.D**	**R1**	**R2**	**R3**	**Average**	**S.D**	**R1**	**R2**	**R3**	**Average**	**S.D**	**R1**	**R2**	**R3**	**Average**	**S.D**
**Me-OH**	6.0	6.0	6.0	**6.0**	0.00	6.0	6.0	6.0	**6.00**	0.00	6.0	6.0	6.0	**6.00**	0.00	6.0	6.0	6.0	**6.00**	0
**Medium + Trp**	7.1	7.5	7.3	**7.30**	0.20	6.0	6.0	6.0	**6.00**	0.00	7.5	7.6	7.2	**7.43**	0.21	6.0	6.0	6.0	**6.00**	0
**JC 673_ Control**	9.8	10	9.1	**9.63**	0.47	7.0	7.2	7.5	**7.23**	0.25	8.3	8.2	8.3	**8.27**	0.06	6.0	6.0	6.0	**6.00**	0
**JC 673_ Trp**	11.0	11.5	11.2	**11.23**	0.25	8.0	8.5	8.6	**8.37**	0.32	12.6	11.5	11.6	**11.90**	0.61	6.0	6.0	6.0	**6.00**	0

Supplementary Figures

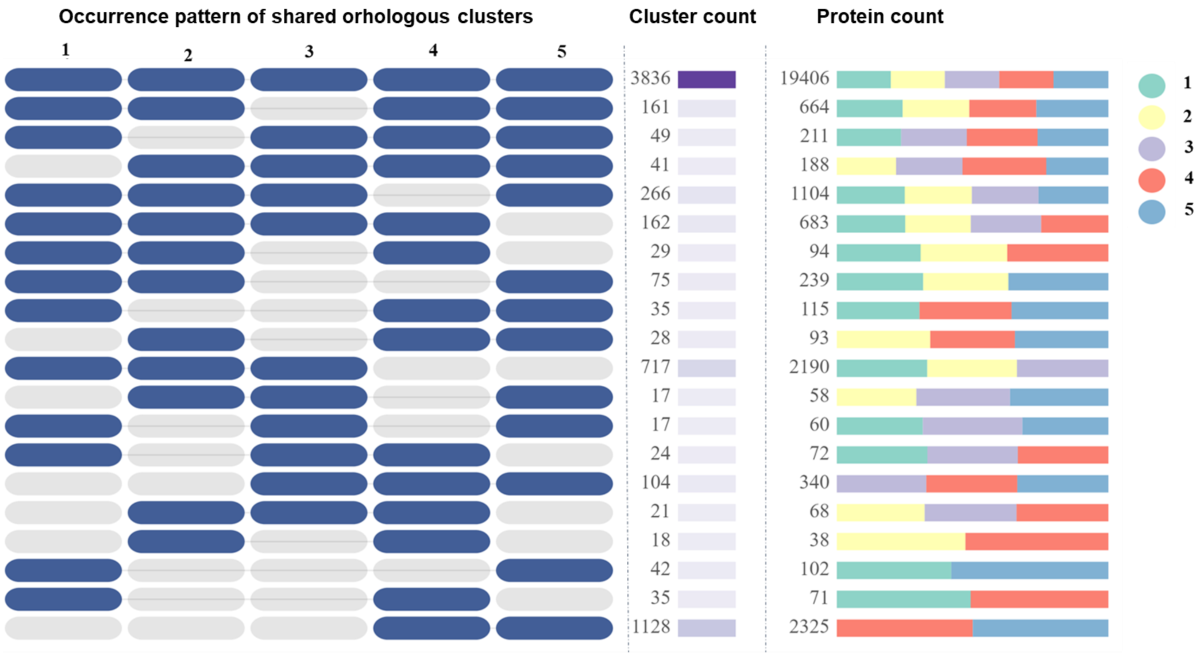

Figure S1. Clusters of orthologous proteins. The occurrence of shared clusters of orthologous proteins in members of the genus *Gemmata* was analysed using the OrthoVenn2 tool. 1: strain JC673^{T}, 2: strain JC717, 3: *G. obscuriglobus* UQM 2246^{T}, 4: *G. palustris* G18^{T}, 5: *G. massilana* IIL30^{T}. Filled boxes indicate the presence of the orthologs clusters, whereas the unfilled boxes indicate their absence. The cluster count corresponding to each row shows the number of shared clusters among species and the protein count is the number of protein families in the corresponding shared clusters which are color-coded for each strain. A number of 3836 clusters is shared among all members of the genus *Gemmata*. 104 clusters are exclusively absent in the new isolates.

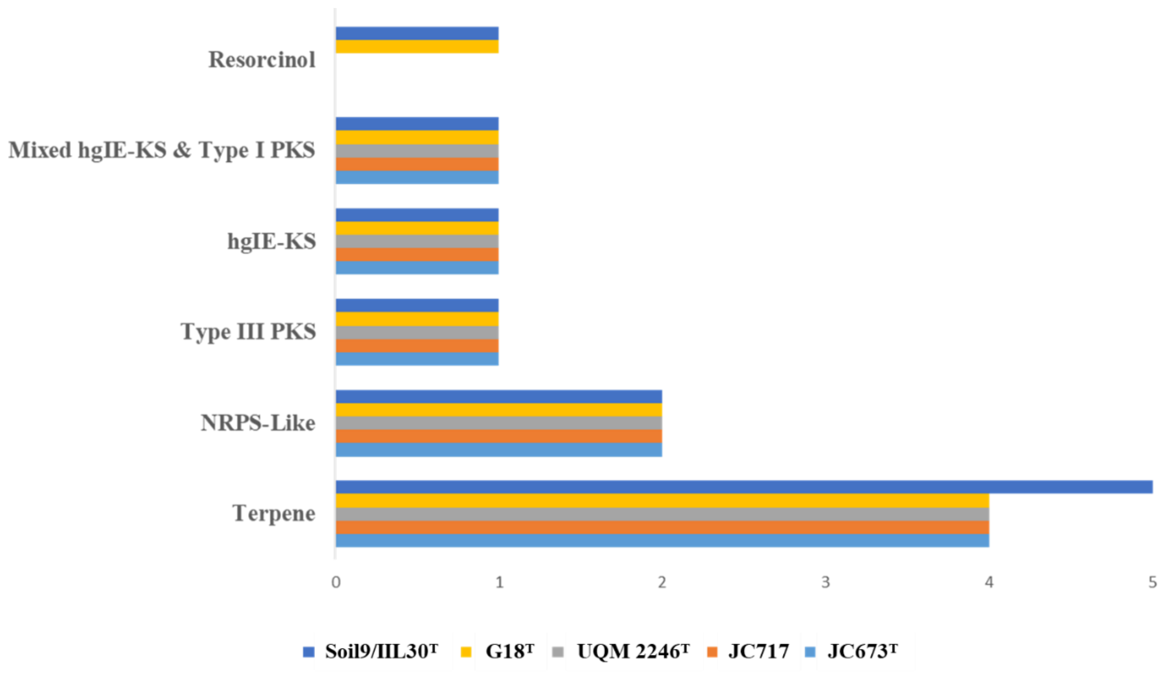

Figure S2. Numbers of biosynthetic gene clusters predicted by antiSMASH. Numbers of predicted genes clusters putatively involved in the biosynthesis of different classes of secondary metabolites based on the genomes of strains JC717, JC673^{T}, *G. obscuriglobus* UQM 2246^{T}, *G. palustris* G18^{T}, and *G. massiliana* IIL30^{T}.

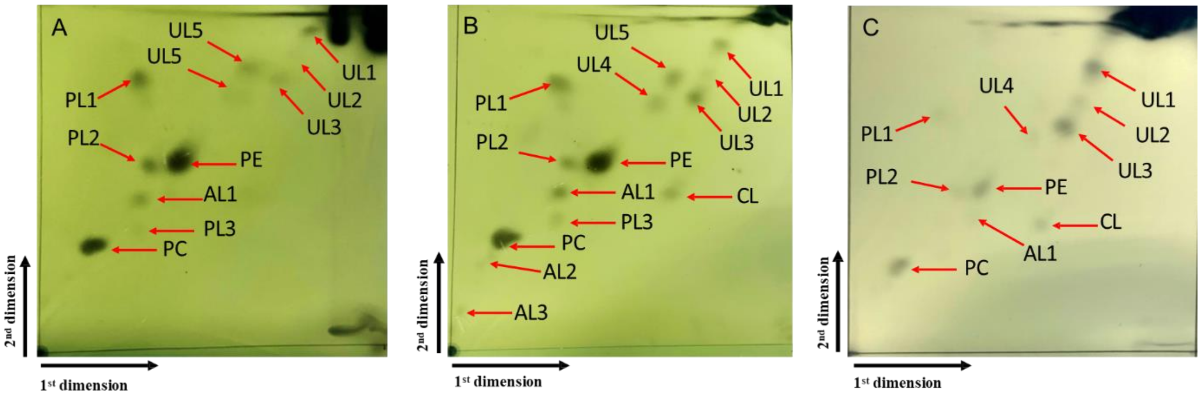

Figure S3. Analysis of polar lipids. Two dimensional chromatograms showing polar lipids of strain JC673^T (A), JC717 (B) and *G. obscuriglobus* UQM 2246^T (C). PC: phosphatidylcholine, PE: phosphatidylethanolamine; AL: unidentified amino lipids, UL: unidentified lipids, PL: unidentified phospholipid, CL: unidentified choline lipid.

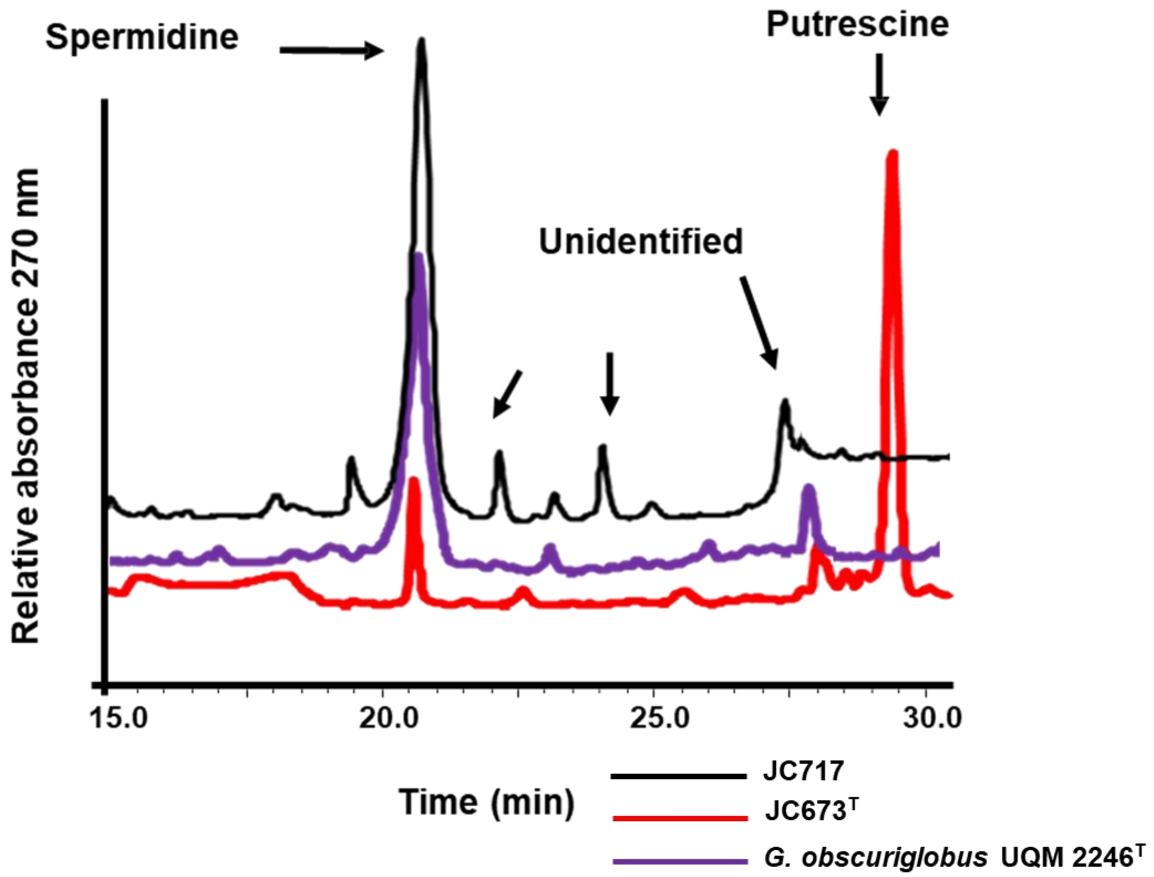

Figure S4. Analysis of polyamines. HPLC chromatograms showing polyamines of strains JC673^T, JC717 and *G. obscuriglobus* UQM 2246^T.

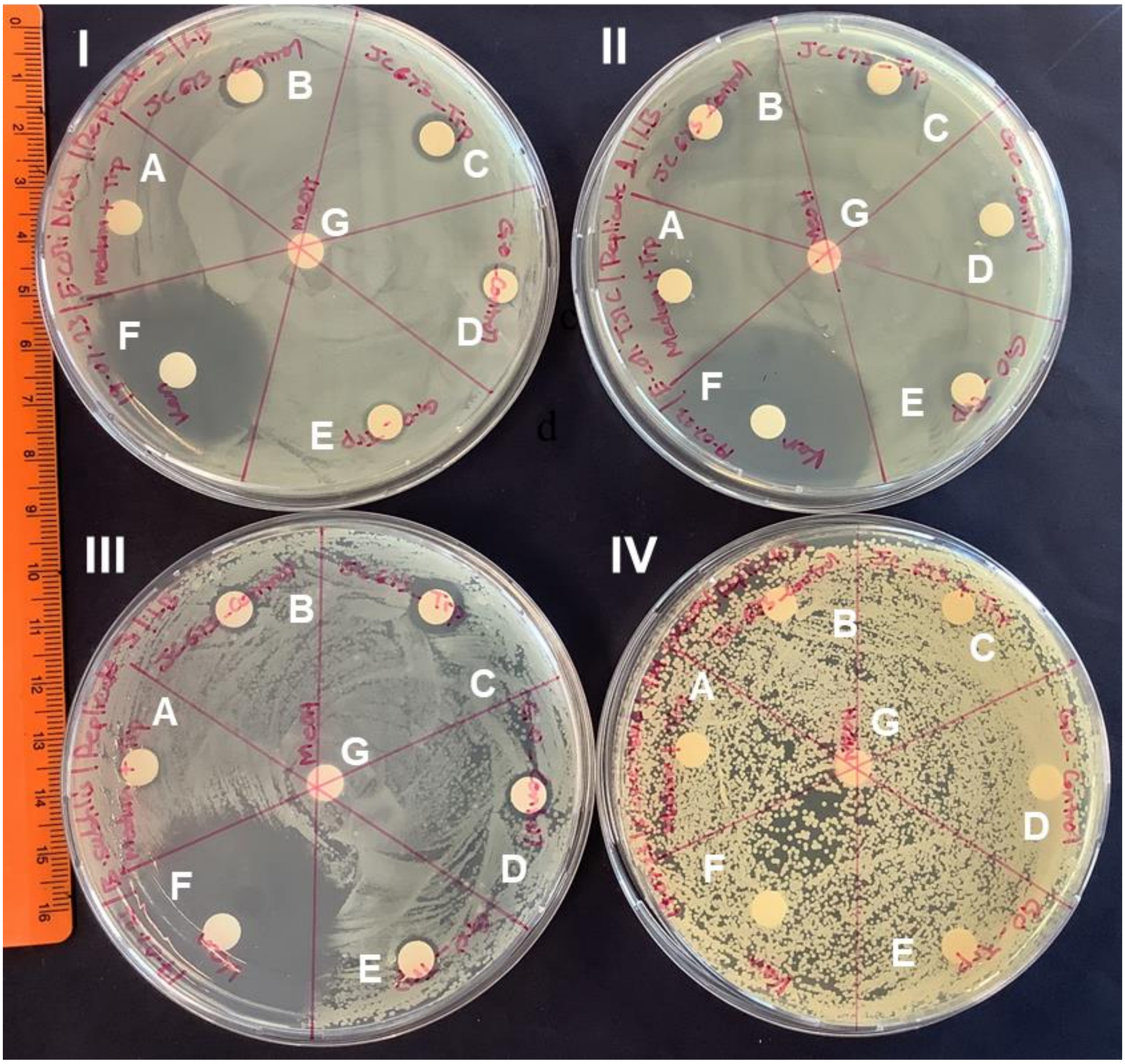

Figure S5. Agar disc diffusion assays for testing antimicrobial activity. Growth inhibition zones for test cultures of (I) *Escherichia coli* DH5α, (II) *Escherichia coli* TolC (a *tolC*-deficient strain), (III) *Bacillus subtilis* and (IV) *Saccharomyces cerevisiae*. Ethyl acetate extracts of cultures of strain JC673^T and *G. obscuriglobus* UQM 2246^T (= DSM 5831^T) along with the respective negative and positive controls were tested. A: extract of medium supplemented with L-Trp (medium only negative control), B: extract of a strain JC673^T culture without L-Trp supplementation, C: extract of a strain JC673^T culture with L-Trp supplementation, D: extract of a *G. obscuriglobus* UQM 2246^T culture without L-Trp supplementation, E: extract of a *G. obscuriglobus* UQM 2246^T culture with L-Trp supplementation, F: kanamycin (1 mg/ml, positive control), G: methanol (resuspension solvent negative control). The photographs were taken after 48 hours of incubation. The experiment was performed in three biological replicates.

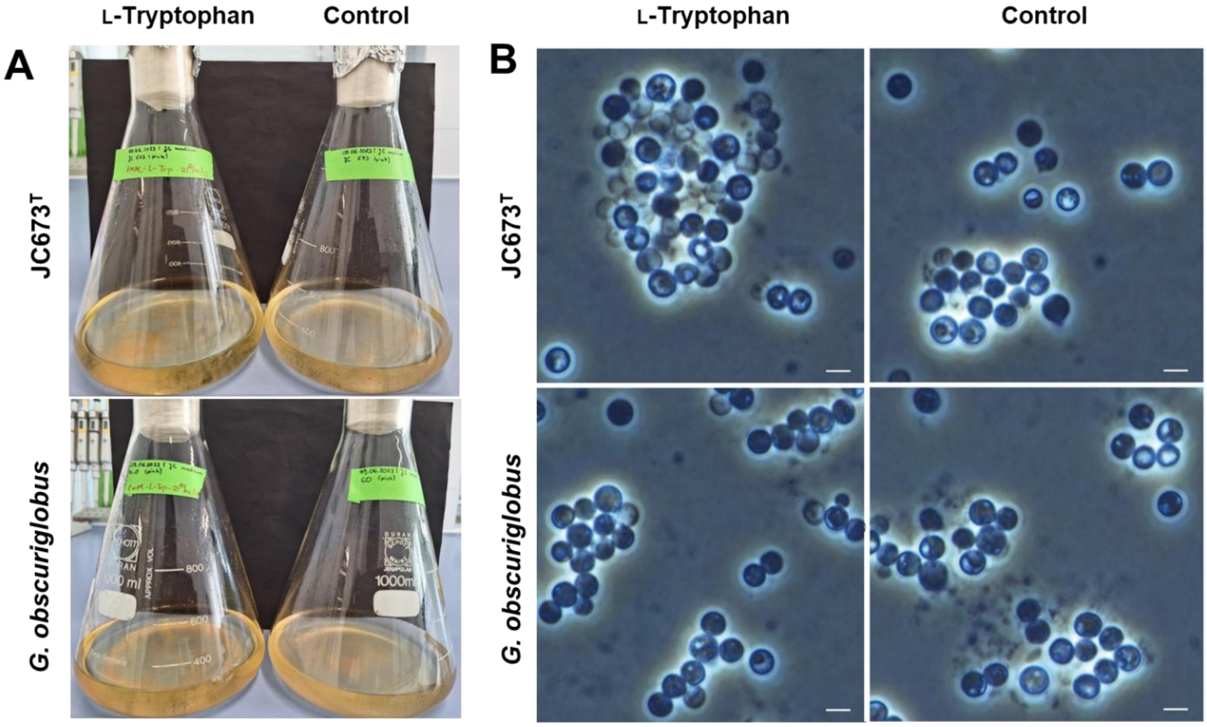

Figure S6. Phenotypical analysis of stationary cultures. (A) Liquid cultures and (B) microscopic visualization of stationary cultures of strain JC673^T and *G. obscuriglobus* UQM 2246^T with and without supplemented L-tryptophan. Both strains form similar round cell under both tested conditions. Occasionally, cells are still budding despite the late stationary phase of the culture. Scale bars represent 2 µm.
